# The flavanone homoeriodictyol increases SGLT-1-mediated glucose uptake but decreases serotonin release in differentiated Caco-2 cells

**DOI:** 10.1371/journal.pone.0171580

**Published:** 2017-02-13

**Authors:** Barbara Lieder, Julia Katharina Hoi, Ann-Katrin Holik, Katrin Geissler, Joachim Hans, Barbara Friedl, Kathrin Liszt, Gerhard E. Krammer, Jakob P. Ley, Veronika Somoza

**Affiliations:** 1 Christian Doppler Laboratory for Bioactive Aroma Compounds, Faculty of Chemistry, University of Vienna, Vienna, Austria; 2 Department for Nutritional and Physiological Chemistry, Faculty of Chemistry, University of Vienna, Vienna, Austria; 3 Symrise AG, Mühlenfeldstraße 1, Holzminden, Germany; George Washington University, UNITED STATES

## Abstract

Flavanoids and related polyphenols, among them hesperitin, have been shown to modulate cellular glucose transport by targeting SGLT-1 and GLUT-2 transport proteins. We aimed to investigate whether homoeriodictyol, which is structurally related to hesperitin, affects glucose uptake in differentiated Caco-2 cells as a model for the intestinal barrier. The results revealed that, in contrast to other polyphenols, the flavanon homoeriodictyol promotes glucose uptake by 29.0 ± 3.83% at a concentration of 100 μM. The glucose uptake stimulating effect was sensitive to phloridzin, but not to phloretin, indicating an involvement of the sodium-coupled glucose transporter SGLT-1, but not of sodium-independent glucose transporters (GLUT). In addition, in contrast to the increased extracellular serotonin levels by stimulation with 500 mM D-(+)-glucose, treatment with 100 μM homoeriodictyol decreased serotonin release by –48.8 ± 7.57% in Caco-2 cells via a phloridzin-sensitive signaling pathway. Extracellular serotonin levels were also reduced by –57.1 ± 5.43% after application of 0.01 μM homoeriodictyol to human neural SH-SY5Y cells. In conclusion, we demonstrate that homoeriodictyol affects both the glucose metabolism and the serotonin system in Caco-2 cells via a SGLT-1-meditated pathway. Furthermore, the results presented here support the usage of Caco-2 cells as a model for peripheral serotonin release. Further investigations may address the value of homoeriodictyol in the treatment of anorexia and malnutrition through the targeting of SGLT-1.

## Introduction

Glucose uptake from the lumen into the epithelial cells of the small intestine is predominantly mediated by the sodium-coupled transporter SGLT-1 [1]. Further transport from the enterocytes to the blood stream is thought to be mediated by the facilitative uniporter glucose transporter 2 (GLUT-2), which exhibits, compared to SGLT-1, a low affinity, but high capacity for glucose [2]. However, in mice [3] and also in human intestinal Caco-2 cells [4], GLUT-2 has been convincingly shown to be expressed not only on the basolateral side, but in the brush-border membranes as well. In addition, more recent studies suggest that at high luminal glucose concentrations, GLUT-2 may be recruited from intracellular vesicles into the apical membrane to support a rapid transport of large quantities of glucose from the lumen to the enterocytes [5]. This recruitment to the brush-border membrane was also demonstrated in Caco-2 cells, demonstrating that this cell line is a suitable model to study intestinal glucose uptake [6, 7].

SGLT-1 has been in the focus of research not only for its role in glucose uptake, but also for its involvement in intestinal nutrient sensing. In addition to its major function as a glucose transporter, SGLT-1 plays a role in intestinal glucose sensing and has been associated with the initiation of gut hormone release [8]. For example, enterochromaffin BON cells have been shown to release the neurotransmitter serotonin in response to D-glucose via a phloridzin-sensitive pathway, pointing to an involvement of SGLT-1 [9]. There is some evidence indicating interaction of serotonin and glucose metabolism although the results are not consistent. However, on the cellular level, serotonin has been shown to increase glucose uptake in L6 myotubes and isolated rat muscle cells [10]. More recent studies demonstrate that intestinal glucose sensing not only involves SGLT-1, but also the sweet taste receptor subunit TAS1R3 and α-gustducin [11].

Flavonoids and related polyphenols have been shown to modulate intestinal glucose uptake by targeting either SGLT-1 or GLUT-2 [12]. On the cellular level, Johnston *et al*. [13] demonstrated that flavonoids potently reduce intestinal glucose uptake by Caco-2 cells. Differences in the inhibitory potential of the compounds in the presence or absence of sodium ions indicate that some compounds have an impact on facilitated glucose transport, while other compounds reduce the sodium-coupled glucose transport. In addition, the flavanone hesperitin has been shown to decrease basal glucose uptake in monocytic U937 cells [14] and MDA-MB-231 breast cancer cells [15]. However, effects of the bitter-masking flavanone homoeriodictyol (HED), which differs from hesperitin only in the position of the -OH and -OCH_3_ residues on the B-ring, on intestinal glucose uptake have not been addressed so far. In the present study, we aimed to investigate whether the polyphenol HED present in *Yerba Santa* [16] modulates intestinal glucose uptake in differentiated Caco-2 cells in a similar way as structurally related polyphenols. Co-incubation studies with inhibitors were used to identify SGLT-1 as the predominant target of HED-mediated glucose uptake. Glucose sensing by SGLT-1 has been associated with an increased release of serotonin in enterochromaffin BON cells [9]. Caco-2 cells have been shown to express the serotonin transporter (SERT) and guanylin as markers for enterochromaffin cells [17] and to release serotonin in response to nutrients [18]. Therefore, this study also focused on the effect of homoeriodictyol, a flavonoid that has not yet been studied for its effects on metabolic pathways involving glucose and serotonin, on Caco-2 cells as a model for intestinal glucose transport and peripheral serotonin release.

## Materials and methods

### Materials

Homoeriodictyol (HED) and its sodium salt (NaHED) were kindly provided by Symrise AG (Germany). The cell lines Caco-2 and SH-SY5Y were purchased from the American type culture collection (ATCC). All other chemicals and reagents were obtained from Sigma Aldrich (Austria), unless stated otherwise.

### Cell culture

Caco-2 cells were cultured under standard conditions (37°C, 5% CO_2_, humidified atmosphere) and differentiated to an enterocyte model within 21 days as described before (18,19). Cells were differentiated between passages 13 and 22 and used for the assays on day 21 ± 3 after seeding. SH-SY5Y cells were cultured under the stated standard conditions as described before (20).

HED and phloridzin were pre-dissolved in ethanol and phloretin was pre-dissolved in DMSO (each 1000 × stock solution). The final concentration of the solvents used for incubation never exceeded 0.1%. The sodium salt of HED was directly dissolved in the incubation media.

### Cell viability

To exclude inhibiting effects of the tested compounds on the metabolic activity of the cells, in the applied test concentrations, cell proliferation, as a measure for cell viability, was determined using the MTT assay as described before (18,19). Treatment of Caco-2 or SH-SY5Y with up to 100 μM HED with or without addition of the inhibitors phloretin (up to 2 mM) and phloridzin (up to 1 mM) did not significantly (*p* > 0.05) reduce metabolic activity (data not shown).

### Glucose uptake

Glucose uptake by differentiated Caco-2 cells was determined using the fluorescently-labeled glucose analog 2-NBDG (2-(*N*-(7-nitrobenz-2-oxa-1,3-diazol-4-yl)amino)-2-deoxyglucose) (Thermo Fisher Scientific, USA) in a 96-well format as described before (18,19). Cells were starved in DMEM lacking D-(+)-glucose, L-glutamine, FBS and phenol red for 60 min, followed by the addition of the test compounds dissolved in Hank’s balanced salt solution (HBSS) containing 20 mM HEPES. After 30 min pre-incubation with the test compounds at 37°C, 2-NBDG was added to a final concentration of 200 μM and incubated for another 30 min at 37°C. Cells were subsequently placed on ice and washed four times with ice-cold PBS before determination of the fluorescence at 480 nm excitation and 550 nm emission. Glucose uptake was quantified relative to untreated control cells in %.

### Determination of intracellular cAMP levels

For determination of intracellular cAMP, cells were treated with HED (100 μM) with or without addition of 500 μM phloridzin, or caffeine (3 mM) and/or forskolin 10 μM as positive controls for 5 min after starving of the cells in DMEM without D-(+)-glucose, L-glutamine, FBS and phenol red for 60 min according to the glucose uptake experiments. The Caco-2 cells were subsequently harvested in lysis buffer (R&D Diagnostics, cAMP ELISA Kit) and lysed by three freeze-thawing cycles with liquid nitrogen. Cellular debris was removed by centrifugation (600 × g, 4°C, 10 min) and the supernatant was analyzed for the cAMP content using the R&D Diagnostics ELISA Kit according to manufacturer’s protocol. cAMP levels were calculated as % of untreated control cells.

### Serotonin release by SH-SY5Y cells and Caco-2 cells

Determination of serotonin levels in the supernatant of SH-SY5Y cells was carried out as described before (20,25). Briefly, cells were seeded in 3.5 cm dishes and stimulated with the HED dissolved in Krebs-Ringer-HEPES buffer (pH 6.2) for 5 min. The serotonin content of the supernatant was quantified using Serotonin sensitive ELISA Kit (DLD Diagnostica, Germany) according to the manufacturer’s protocol. Data are presented as % of untreated or vehicle treated control cells as indicated in the figure legends. Serotonin release by differentiated Caco-2 cells was determined analogously to the procedure described for SH-SY5Y cells, but with slight modifications: Caco-2 cells were differentiated and stimulated in 24-well or 12-well format using Krebs-Ringer HEPES buffer with a pH of 7.4.

### Quantitative real-time PCR

The gene expression level of the sodium-coupled glucose transporter 1, SGLT-1 (*SLC5A1*), after treatment with 100 μM HED for 15, 30 and 60 min, was analyzed using two-step reverse transcription quantitative real-time PCR (qPCR). Total RNA was isolated using the PeqGold Total RNA isolation Kit (Peqlab, Germany) and the quality and concentration were analyzed using a NanoQuant Plate with an Infinite M200 Tecan reader. Reverse transcription was performed by means of high capacity cDNA Kit (Life technologies, Fisher Scientific, Austria) according to manufacturer’s protocol. RT-qPCR was carried out in triplicate on a StepOne Plus device (Applied Biosystems, Fisher Scientific, Austria) with Fast SYBR Green Master Mix (Life technologies, Fisher Scientific, Austria). The following primers were used for selective amplification:

SLC5A1-forward-CCGATATCTCCATCATCGTTATCTAC-5'; SLC5A1-reverse: 3'-CACGATTGGTGGAAAACATAGC-5' (22); HPRT-forward:3'-TGCTCGATGTGATGAAGGAG-5'; HPRT-reverse:3'-ATAGCCCCCCTTGAGCACAC-5' (18,19), GAPDH-forward: 3'-AGGTCGGAGTCAACGGATTTG-5'; GAPDH-reverse: 3'-GGGGTCATTGATGGCAACAATA-5' (designed using PrimerBlast (23)). Hypothetical starting concentrations of the respective mRNA were calculated using LinREG PCR v.12.8 (24) and the fold change to non-treated control cells was determined after normalization to the geometric mean of the two reference genes HPRT and GAPDH.

### Statistics

Data are expressed as fold changes ± SEM from at least three biological replicates with multiple technical replicates, each after removal of outliers according to the Nalimov outlier test. Data sets were tested for normal distribution using the Shapiro-Wilk test. Comparisons between two groups were analyzed using Student’s *t*-test or Mann-Whitney U test for non-normally distributed data sets, respectively. For comparison of multiple groups one-way or two-way ANOVA with Holm-Sidak p*ost hoc* test or ANOVA on ranks, respectively, was applied. All statistical analysis except the Nalimov outlier test were performed using Sigma Plot 11 or 13 (Systat Software, USA). The Nalimov outlier test was carried out using Excel 2007 (Microsoft).

## Results & discussion

**Fig 1:**
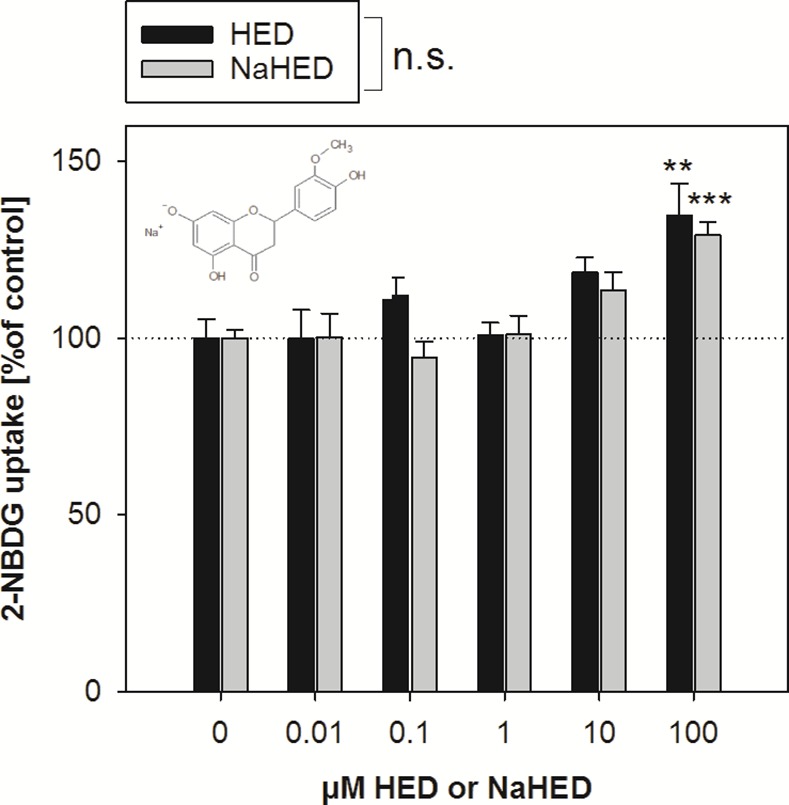
2-NBDG uptake by differentiated Caco-2 cells after 30 min pre-treatment with 0.01 to 100 μM HED or HED sodium salt. Results are calculated in comparison to the corresponding control (incubation buffer for the sodium salt of HED and incubation buffer containing 0.1% EtOH for HED). Statistics: *n* = 4–9 with multiple technical replicates. Significant differences between the concentrations and treatments were assessed using two-way ANOVA with Holm-Sidak *post hoc* test. ** *p*<0.01, *** *p*< 0.001 *vs*. control. n.s.: not significant.

Several polyphenols have been shown to influence intestinal glucose uptake. However, results are inconsistent since a structure-associated activity has not yet been demonstrated; whereas phloretin, myricetin, and quercetin affect glucose uptake *via* GLUT transporters, the polyphenols phloridzin and neohesperidin have been shown to decrease sodium-dependent glucose uptake, indicating a SGLT-1-dependent mechanism [13]. Since the polyphenol hesperitin was demonstrated to decrease glucose uptake in two different cell models [14, 15], we hypothesized here that the structural analog homoeriodictyol (HED) affects glucose uptake in a similar manner. Differentiated Caco-2 cells were chosen since these cells have been demonstrated to express the relevant glucose transport systems, such as SGLT-1 at the apical surface [19] and GLUT-2 at the basolateral and apical surface [5]. Full differentiation within 21 days of cultivation applied in this study has been confirmed in earlier studies using the trans-epithelial electrical resistance as a marker [20, 21]. The test compound HED differs from hesperitin only in the position of the residues at the B-ring. However, in contrast to our hypothesis, this slight structural difference led to a major difference in the impact on glucose uptake. Both HED and its sodium salt instead increased glucose uptake in differentiated Caco-2 cells up to 135 ± 8.89% (*p* = 0.002) and 129 ± 3.83% (*p*<0.001), respectively, after application of the highest test concentration of 100 μM (Fig 1). These data demonstrate that slight structural differences may have a major impact on the bioactivity of a compound and that not all polyphenols reduce glucose uptake. Since higher concentrations could not be applied in the assay due to HED’s limited solubility in aqueous solutions, no saturation point of HED on glucose uptake is presented here. Comparison of the effects of the HED pre-dissolved in ethanol with the more water soluble sodium salt confirmed that there is no difference in the effect size between the two modes of application (*p* = 0.47). Therefore, an impact of the sodium ions on glucose uptake can be excluded. In addition, the use of ethanol during incubations was avoided, and the following experiments in Caco-2 cells were carried out using the water-soluble sodium salt of HED.

The effects of the dietary polyphenols tested by Johnston *et al*. [13] were partly sodium-dependent and partly not, associating the effects to either SGLT-1 or facilitative glucose transporters. Thus, we investigated whether the effects of HED were the result of the activity of one or both of the most prominent intestinal glucose-transport systems, the sodium-dependent SGLT-1 and the sodium-independent GLUTs. For inhibition of SGLT-1, we concomitantly applied phloridzin, and for inhibition of sodium-independent GLUTs, phloretin was used. The concentrations applied for the present study were chosen to cover a broad range, between no effect on glucose uptake itself up to a saturated glucose uptake inhibition. However, the maximum concentration was limited by the low solubility of phloretin (2 mM) and phloridzin (1 mM) in aqueous solutions. An effect of the 0.1% ethanol used as a solvent for phloridzin and 0.1% DMSO applied as a solvent for phloretin was excluded in preliminary experiments and data are calculated and statistics assessed in comparison to the corresponding solvent control.

**Fig 2:**
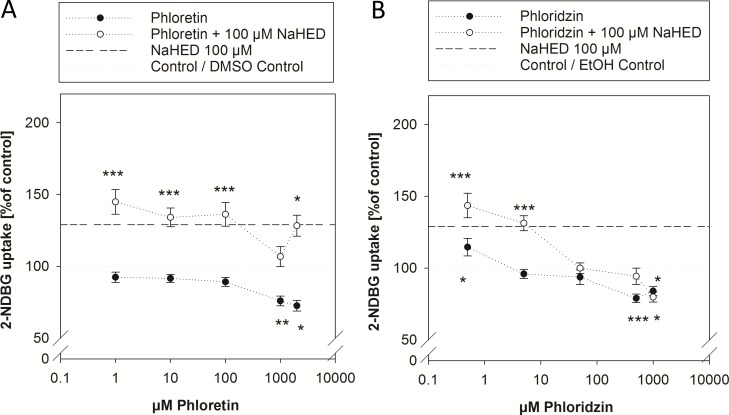
**2-NBDG uptake by differentiated Caco-2 cells after 30 min pre-treatment with 1** μ**M to 2 mM phloretin (2A) or 0.5** μ**M to 1 mM phloridzin (2B) with or without addition of 100** μ**M HED sodium salt**. Data are calculated in comparison to the corresponding solvent control (100%) after excluding an effect of the solvent on glucose uptake. Standard error means of the used controls were 100 ± 6.78% for 0.1% DMSO control (A), 100 ± 4.06% for 0.1% EtOH control (B), and 100 ± 3.85% for the buffer control (not shown). Control values were set to 100% and are depicted using a grey line. Pre-incubation with 100 μM HED sodium salt increased glucose uptake by an average of 29.0 ± 3.90% and is symbolized by a dotted black line. Statistics: *n* = 3–6 with multiple technical replicates. Significant differences between the treatments and control treated cells were assessed using one-way ANOVA *vs*. the corresponding solvent control with Holm-Sidak *post hoc* test and are marked by * *p* <0.05, ** *p*<0.01, *** *p*< 0.001.

Fig 2A demonstrates that application of phloretin decreased glucose uptake in Caco-2 cells at concentrations greater than 1 mM to a maximum extent of –27.5 ±8.89% (*p* = 0.038 *vs*. DMSO control) at 2 mM. Application of the SGLT 1 inhibitor phloridzin resulted in a significant decrease in glucose uptake compared to untreated control cells at concentrations higher than 0.5 mM to a maximum extent of –21.1 ± 4.69% (*p*<0.001 *vs*. EtOH control, Fig 2B). Both inhibitors alone reduced glucose uptake at the higher test concentration, but did not completely abolish glucose uptake. Complete inhibition of glucose uptake by inhibitors of a single glucose transporters has not been demonstrated by other groups either [13], and may be explained by compensation *via* the other glucose transport mechanism and the limitation of applicable concentrations in water-based media.

With exception of an addition of 1 mM phloretin, the glucose uptake promoting effect of 100 μM HED sodium salt was not affected by the addition of 1 μM– 2 mM phloretin (*p*<0.001 *vs*. DMSO control) (Fig 2A). In contrast to the addition of phloretin, the glucose uptake promoting effect of 100 μM HED sodium salt was abolished by concomitant incubation with phloridzin, starting from a concentration of 50 μM (*p* = 0.992 *vs*. EtOH control, Fig 2B). The phloridzin-sensitive pathway indicates that glucose-uptake stimulation by HED sodium salt involves SGLT-1, but not sodium-independent glucose transporters such as GLUT-1 or GLUT-2.

**Fig 3:**
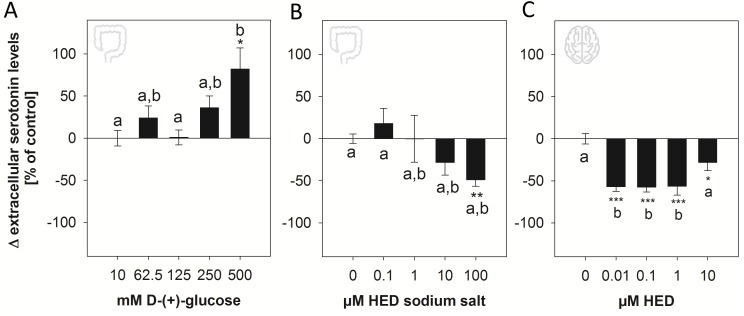
(A) Serotonin levels in the supernatant of differentiated Caco-2 cells after stimulation with 125 to 500 mM D-(+)-glucose for five minutes. (B) Serotonin levels in the supernatant of differentiated Caco-2 cells after stimulation with 0.01–100 μM HED sodium salt for five minutes. (C) Serotonin levels in the supernatant of SH-SY5Y cells after stimulation with 0.001–10 μM HED after five minutes. Krebs-Ringer buffer containing 10 mM D-(+)-glucose was used as control in all studies and was set to 100%, the treatments are depicted as the mean change of extracellular serotonin levels in percent of the control ± SEM. Statistics: *n* = 3–6 with at least two technical replicates. Significant differences between the treatments were assessed using one-way ANOVA with Holm-Sidak *post hoc* test and are marked by * *p* <0.05, ** *p* <0.01, *** *p* <0.001 vs. control and with distinct letters for differences between the groups.

Luminal glucose in the small intestine stimulates the release of several digestive hormones, and also the neurotransmitter serotonin [22]. Mechanistic studies in enterochromaffin BON cells revealed that stimulation of serotonin release by D-(+)-glucose is mediated by a phloridzin-sensitive pathway, suggesting a link to SGLT-1 [9]. Caco-2 cells, although not a model for enterochromaffin cells, have been shown to release serotonin in response to a nutritive stimulus, monooleoylglycerol [18]. We therefore aimed to investigate whether SGLT-1 activation by HED results in increased serotonin levels as well. First, we confirmed that serotonin release by Caco-2 cells responds to D-(+)-glucose similarly to enterochromaffin cells. Serotonin levels in the supernatant of Caco-2 cells were increased by 81.9 ± 25.0% (*p* = 0.024 *vs*. control) in response to the highest test concentration of 500 mM D-(+)-glucose (Fig 3A). In contrast to D-(+)-glucose, treatment with HED sodium salt decreased dose-dependently serotonin levels in the supernatant of Caco-2 cells up to –48.8 ± 7.57% at 100 μM (*p*<0.01 *vs*. buffer control, Fig 3B). Although the glucose concentration needed for a robust increase in serotonin levels in the supernatant of Caco-2 cells is about 10-times higher than in BON cells [9], our finding supports that Caco-2 cells are a suitable model to study serotonin release in response to nutritive stimuli, especially hexoses. However, in contrast to our hypothesis, stimulation with HED did not increase, but instead decreased extracellular serotonin levels of Caco-2 cells. This result was confirmed using a human neuronal cell model, SH-SY5Y cells, which has been successfully used to study serotonin release [23, 24]. (Fig 3C). Only concentrations up to 10 μM HED were applied, since higher concentrations are very unlikely to be reached in the central nervous system. In accordance with the results obtained with Caco-2 cells, stimulation with 0.01–10 μM HED led to a decrease in serotonin levels in the supernatant of neural SH-SY5Y cells by up to –57.1 ± 5.43% at 0.01 μM (*p*< 0.001 *vs*. control), suggesting that HED may also act on the central serotonin system. The similar response of Caco-2 and SH-SY5Y cells not only supports Caco-2 cells as a suitable model for serotonin release, but also points to parallel mechanisms of central and peripheral serotonin release. However, similarities in the mechanistic pathways involved in the response of the central and peripheral serotonin systems need to be investigated in depth in future studies.

As a next step, the question whether SGLT-1 links glucose uptake with serotonin release in Caco-2 cells was addressed. We analyzed serotonin levels in the supernatant of Caco-2 cells after treatment with glucose and HED sodium salt in combination with phloridzin at 5, 50 and 500 μM to cover the whole range between no effect on glucose uptake and reduction of glucose uptake by inhibition of SGLT-1. An impact on serotonin levels of a treatment with 5–500 μM phloridzin solely was excluded (*p*>0.05 *vs*. EtOH control, data not shown).

**Fig 4:**
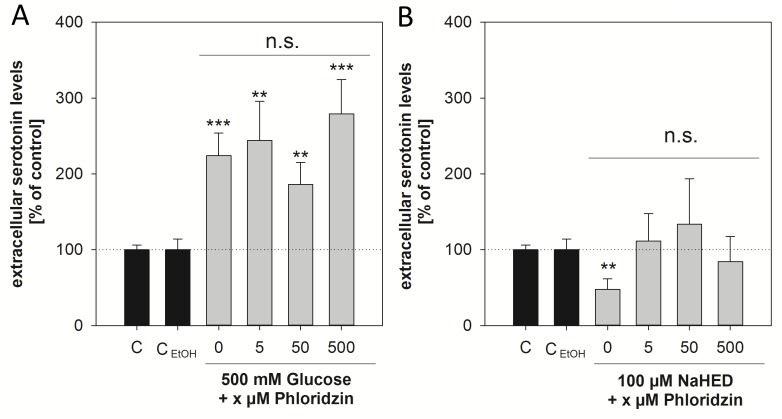
A, B: Extracellular serotonin levels of differentiated Caco-2 cells after stimulation with 500 mM D-(+)-glucose (A) or 100 μM HED sodium salt (B) with or without addition of 5–500 μM phloridzin. Krebs-Ringer buffer without, or in case of incubations using phloridzin, with addition of 0.1% EtOH was used as control and set to 100%. An effect of 0.1% EtOH was excluded in preliminary studies. Statistics (A, B): *n* = 3 with two technical replicates. Significant differences between the treatments were assessed using one-way ANOVA with Holm-Sidak *post hoc* test and are marked by n.s. (not significant), whereas significant differences to the controls are marked with * *p* <0.05, ** *p* <0.01, *** *p* <0.001 *vs*. the corresponding control (incubations using phloridzin were tested in comparison to the EtOH control, treatments with glucose or HED sodium alone were tested in comparison to the incubation media control).

In contrast to the results of Kim *et al*. [9] in BON cells, extracellular serotonin levels after stimulation with glucose in Caco-2 cells were not sensitive to phloridzin, as the concomitant treatment of Caco-2 cells with 500 mM D-(+)-glucose in combination with 5–500 μM phloridzin was not different from a treatment with 500 mM D-(+)-glucose alone. Additionally, the stimulating effect of 500 mM D-(+)-glucose, compared to non-treated control cells, was not reduced (*p*<0.05 *vs*. EtOH control) (Fig 4A). Therefore, a SGLT-mediated pathway for glucose in serotonin release is not assumed and demonstrating that glucose uptake is not generally linked to serotonin release via SGLT-1 in Caco-2 cells. In addition, the concentration of glucose needed to induce an increase in serotonin levels is more than 10-times higher than the saturating concentration of SGLT-1 (~ 40 mM) [25], which is also supporting the proposed SGLT-1-independent pathway. However, the effect of HED on serotonin release was reduced by the addition of phloridzin: treatment with 100 μM HED sodium salt in combination with phloridzin did not lead to decreased serotonin levels in the supernatant compared to control treated cells (*p*>0.05 *vs*. EtOH control) as a treatment with only 100 μM HED sodium salt did. (Fig 4B). This suggests that SGLT-1 not only plays a role in HED-mediated glucose uptake, but also in the here demonstrated decreased serotonin levels in the supernatant. Also, in Caco-2 cells, SGLT-1 activation is not invariably associated with an increased serotonin release, but may contribute to decreased serotonin levels as well.

**Fig 5:**
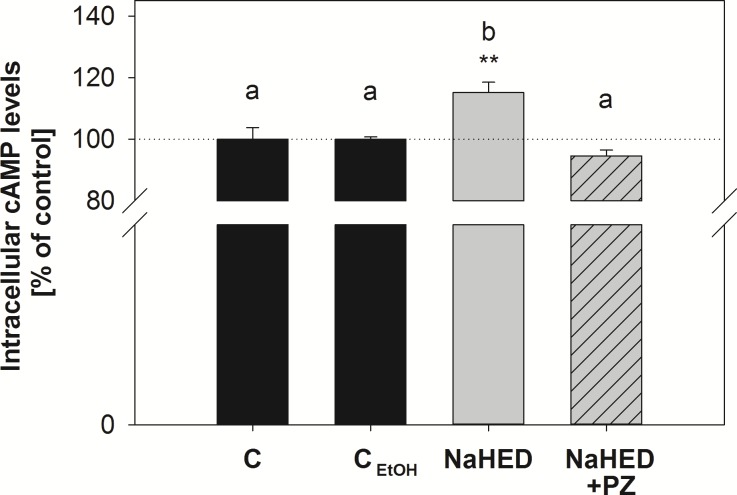
Intracellular cAMP levels after 5 min incubation with 100 μM HED sodium salt with or without addition of 500 μM phloridzin (PZ). Statistics: Mean % of control levels ± SEM from three independent experiments with two technical replicates. Significant differences between the treatments were assessed using one-way ANOVA with Holm-Sidak *post hoc* test and are marked with * *p* <0.05, ** *p* <0.01, *** *p* <0.001 *vs*. the corresponding control (incubations using phloridzin were tested in comparison to the EtOH control, treatments with glucose or HED sodium alone were tested in comparison to the incubation media control) or n.s.: not significant.

Current literature supports a connection of intracellular cAMP levels and SGLT-1. [26]. Thus, as a signaling pathway, we hypothesized that HED impacts SGLT-1-mediated serotonin levels and glucose uptake via the cAMP / proteinkinase A pathway. Intracellular cAMP levels were determined after 5 min stimulation with the positive controls, the phosphodiesterase-inhibitor caffeine [26], or the adenylate-cyclase stimulant forskolin [27], and the test compound HED with or without addition of phloridzin. The positive controls, 3 mM caffeine and 10 μM forskolin, comparably increased intracellular cAMP levels to 141 ± 8.73% (*p*<0.001) and 132 ± 8.67% (*p*<0.05, one-way ANOVA with Holm-Sidak *post-hoc* test, *n* = 3–6). A signaling of HED via the cAMP / proteinkinase A-pathway is supported by an increase in intracellular cAMP levels to 115 ± 3.32%, (*p*< 0.01 *vs*. control and EtOH control) after a five minute-stimulation with 100 μM HED (Fig 5).

Moreover, addition of the SGLT-1 inhibitor phloridzin to HED-containing incubation medium slightly diminished the stimulating effect of HED to 94.6 ± 1.87% of the control (*p*>0.05 *vs*. control or control with 0.1% EtOH; p<0.001 vs. HED, one-way ANOVA) (Fig 5). This finding further supports a signaling for HED *via* SGLT-1 followed by a cAMP-dependent pathway. An increase of SGLT-1 abundance in Caco-2 cells after the short-term incubations with HED is unlikely since no difference in mRNA levels of *SLC5A1*, encoding for SGLT-1 was detected after incubation with 100 μM for 15, 30 and 60 min. The fold changes in comparison to non-treated controls were 1.09±0.24 after 15 min, 0.85±0.07 after 30 min and 0.94±0.02 after 60 min (*p*>0.05 for all treatments, one-way ANOVA). Therefore, we hypothesize an increase in SGLT-1 activity rather than an increase in SGLT-1 abundance caused by HED treatment. This activation may not only affect extracellular serotonin levels, but also glucose uptake after 30 min pre-incubation. However, both systems are not generally linked in Caco-2 cells, since glucose-mediated serotonin release is not SGLT-1-dependent.

For glucose, it is conceivable that, instead of an intracellular activation *via* cAMP, signaling *via* membrane-bound sweet taste receptor TAS1R3 and α-gustducin, as in enterocyte nutrient sensing [27], may lead to increased serotonin release. The release of neuropeptides and their receptor binding may increase cAMP in the long-term as well [8], involving SGLT-1 in a later stage of the signaling pathway, but not after short-term incubations like in serotonin release. A pathway involving TAS1R3 and α-gustducin for glucose might at least partially explain the contrary effects of glucose and HED and the non-phloridzin-sensitive response of glucose in serotonin release, but needs to be clarified in future studies as well. Moreover, since serotonin levels in the supernatant were not altered after treatment with 500 mM of the non-transported sugar D-mannitol, with a mean value of 121±19.0% in comparison to the control (*p*>0.05), it can also be excluded that osmotic action has influence on extracellular serotonin levels after stimulation with 500 mM glucose. In addition, it has to be noticed that the glucose uptake assay in its present form is a model for luminal glucose uptake only. To evaluate the impact of homoeriodictyol transport mechanisms regulating blood glucose levels in humans, transport from the enterocytes to the blood side as well the effect of HED on glucose uptake in peripheral muscle cells and adipocytes would have to be considered as well. This approach will be considered in future *in vivo* studies.

## Conclusion

In conclusion, we demonstrate that the polyphenol homoeriodictyol affects both glucose metabolism and the serotonin system in Caco-2 cells *via* a SGLT-1-meditated pathway. Since stimulation of serotonin levels by glucose is, unlike reduced serotonin levels after HED treatment, not phloridzin-sensitive, a direct link between of glucose uptake and serotonin release *via* SGLT-1 in Caco-2 cells is not assumed. However, the results presented here support the usage of Caco-2 cells as a model for peripheral serotonin release.
